# ‘I was selected to be trained as an aid post orderly’

**DOI:** 10.1098/rstb.2008.4032

**Published:** 2008-11-27

**Authors:** Tarubi Taguse

**Affiliations:** Purosa, c/o Papua New Guinea Institute of Medical ResearchPO Box 60, Goroka, EHP 441, Papua New Guinea

The administrator of the district, Mr John Colman, first employed me as a translator assisting the late Mr Muriso Warebu. I assisted him and in particular worked with the Gimi people and the people of Moraei. Then I was selected to be trained as an aid post orderly. After completing the course, I was sent back to Purosa to set up an aid post. With the help of the village people, the building was ready in less than two weeks.

During my working days, I visited and cared for more patients with kuru than with any other disease. I also assisted Dr Carleton Gajdusek in performing autopsies and stitching up the body and handing it back to the family for burial. Kuru had devastating social effects: I can remember one hamlet in the Purosa Valley where at one stage there were only five women with many more men and children.

Carleton was based in Agakamatasa village and I went out to help and treat patients in that area too. I also worked with Michael Alpers, Dr Hornabrook, John Mathews, Dick Sorenson and Paul Brown. I am particularly pleased that some of those I worked with in the past have been able to attend this meeting.

The medical scientists who went into Okapa District from abroad played a vital role in reducing the death rate of kuru disease. They helped to change the lifestyle of our people. We have lost many of our family members from kuru, which swept through entire villages taking innocent lives. However, we have recovered and today the population of the Purosa Valley is nearly 4000. Yet I still have one unanswered question: has kuru disease gone for good or will it return in the next generation?

